# Plant Defense Proteins: Recent Discoveries and Applications

**DOI:** 10.3390/plants14132069

**Published:** 2025-07-06

**Authors:** Samuel O. Shobade, Marit Nilsen-Hamilton, Olga A. Zabotina

**Affiliations:** 1 Ames National Laboratory, U. S. Department of Energy, Ames, IA 50011, USA; marit@iastate.edu; 2 Roy J. Carver Department of Biochemistry, Biophysics and Molecular Biology, Iowa State University, Ames, IA 50011, USA

**Keywords:** plant defense, pathogenesis-related proteins, chitinases, chitin-binding domain, rhizosphere, jacalin-related lectins, G-proteins, β-glucosidase

## Abstract

Proteins play pivotal roles in safeguarding plants against numerous biotic and abiotic stresses. Understanding their biological functions and mechanisms of action is essential for advancing plant biology, agriculture, and biotechnology. This review considers the diversity and potential applications of plant defense proteins including pathogenesis-related (PR) proteins, chitinases, glucanases, protease inhibitors, lectins, and antimicrobial peptides. Recent advances, such as the omics technologies, have enabled the discovery of new plant defense proteins and regulatory networks that govern plant defense responses and unveiled numerous roles of plant defense proteins in stress perception, signal transduction, and immune priming. The molecular affinities and enzymatic activities of plant defense proteins are essential for their defense functions. Applications of plant defense proteins span agriculture, biotechnology, and medicine, including the development of resistant crop varieties, bio-based products, biopharmaceuticals, and functional foods. Future research directions include elucidating the structural bases of defense protein functions, exploring protein interactions with ligands and other proteins, and engineering defense proteins for enhanced efficacy. Overall, this review illuminates the significance of plant defense proteins against biotic stresses in plant biology and biotechnology, emphasizing their potential for sustainable agriculture and environmental management.

## 1. Introduction

Throughout their evolutionary history, plants have developed multi-level passive and active resistance mechanisms that they may employ in concert to fend off pathogen invasion [1,2]. Physical barriers on the plant cell surface and molecules poisonous to pathogens within the cell—such as antibacterial agents, phenols, unsaturated lactones, and antimicrobial peptides—are critical components of passive resistance [3]. After a pathogen infection, the body quickly initiates active defense, which includes forming phytoalexin, releasing reactive oxygen species (ROS), the hypersensitive response (HR), and reinforcing and repairing the cell wall [3,4].

This review aims to provide an overview of plant defense proteins, focusing on their classifications, applications, and recent discoveries on their localization, structure, and function. By synthesizing the latest research findings and insights, we seek to elucidate the multifaceted roles of plant defense proteins in plant biology, agriculture, and biotechnology and to outline future directions for research and innovation in this rapidly evolving field.

## 2. Plant Stressors and Defense Strategies

### 2.1. Pathogens

**Figure 1:**
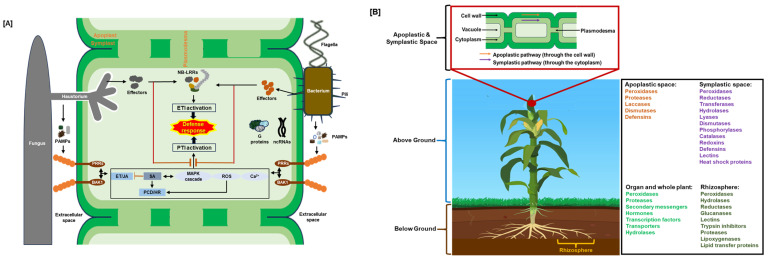
Plant immune system and defense proteins. (**A**) Schematic of plant’s innate immune system: ETI, effector-triggered immunity; HR, hypersensitive response; JA/ET, jasmonic acid/ethylene; MAPK, mitogen-activated protein kinase; NB-LRR, nucleotide-binding leucine-rich repeat; ncRNAs, non-coding RNAs; PAMPs, pathogen-associated molecular patterns; PCD, programmed cell death; PRRs, pattern recognition receptors; PTI, PAMP-triggered immunity; ROS, reactive oxygen species; SA, salicylic acid. Positive regulation is shown by black arrows, and negative regulation is indicated by red open blocks. (**B**) Localizations of plant defense proteins. Plant defense proteins function at the cellular, organ, and whole-plant levels to stop or lessen the spread of stress effects inside the plant. These functions include stomatal control and hypersensitive and systemic responses [8,9,10]. Additionally, defense can occur at the ecosystem level by releasing volatile organic chemicals into the rhizosphere and aboveground environment [11,12] and exuding proteins and signaling molecules from the roots [13].

When pathogens like bacteria, fungi, oomycetes, mycoplasma, or viruses attack, the plant defense response is carefully controlled. Four stages of pathogen-plant interactions have been defined, with PAMP- or pattern-triggered immunity (PTI) and effector-triggered immunity (ETI) considered the main defense mechanisms [5,6,7]. In the first stage, transmembrane pattern recognition receptors (PRRs) identify motifs on pathogens known as pathogen-associated molecular patterns (PAMPs) (such as proteins/peptides, polysaccharides, lipopolysaccharides (LPS), peptidoglycans (PGNs), and nucleic acids) and molecules originating from the sites of damage (damage-associated molecular patterns (DAMPs)) initiate the initial stage of an immune response (Figure 1). Through molecular cascades, receptors convey the signal that results in the expression of PR genes related to pathogenesis [3,4]. The PTI response can be enough to prevent pathogens from colonizing plant cells. Upon identifying the PAMPs generated by pathogens, resistant hosts initiate PTI responses, such as calcium ion (Ca^2+^) influx, generation of reactive oxygen species (ROS), and activation of mitogen-activated protein kinase (MAPK). Subsequent activation of the downstream salicylic acid (SA) or jasmonic acid (JA)/ethylene (ET) signal pathway triggers the synthesis of defense components that result in plant disease resistance (Figure 1).

In the second stage of infection, pathogens deliver effectors such as AvrPto, AvrPtoB, and AvrPphB, which disrupt PR gene expression or downstream action. These effectors are inserted into the plant cells via the pathogen type III secretion system. This series of events cumulatively contributes to effector-triggered susceptibility (ETS), which is the third stage in plant–pathogen interactions. The effectors can spread throughout the infection site and feed on the plant’s tissues, rendering the plant susceptible to disease [14].

Effector-triggered immunity is initiated in the third stage of infection when the effector molecules of the pathogen are recognized by internal receptors of the host, such as receptors with nucleotide-binding domains and leucine-rich repeats (NB-LRR). These proteins are encoded by a group of resistance (R) genes that encode receptors and mostly recognize pathogen effectors by direct interaction [15]. The ETI is an enhanced version of the PTI response that often results in a hypersensitivity response (HR), which is characterized by localized programmed cell death (PCD) at the point of pathogen attack, and systemic acquired resistance (SAR) that involves signal generation, salicylic acid (SA) accumulation, and pathogenesis-related (PR) protein production, which is similar to the innate immune system in animals [16]. As in stage one, this reaction may be sufficient to provide resistance.

By either gaining more effectors or eclipsing the receptors for pathogen identification, the pathogen escapes ETI in the fourth stage by evolving new effectors (that evade recognition by host R proteins) through various genetic mechanisms such as gene mutation and diversification. An example is seen in *Pseudomonas syringae*, as they evade recognition by tomato Pto kinase by evolving altered AvrPtoB effectors [17]. Another strategy is effector gene duplication and recombination, such as in rust fungi (*Puccinia* spp.), which create new virulence alleles via recombination to evade wheat’s R genes [18]. Yet another strategy of pathogen escape is hiding effectors or reducing their expression, as seen with Phytophthora infestans (potato late blight pathogen), which reduces effector expression to evade recognition by potato R genes [19]. These pathogen responses occur due to selection pressure during the plant–pathogen co-evolutionary arms race involving R genes and Avr effectors [20]. The gene-for-gene model provides a more detailed explanation of the mechanisms behind effector-triggered immunity, including the interplay between plant R genes and the pathogen’s sole dominant avirulence gene (*Avr*). Recognition of pathogen effectors by plant resistance (R) proteins initiates an incompatible interaction, often mediated by NB-LRR receptors. Upon effector recognition, these receptors undergo conformational changes that activate downstream defense signaling pathways, triggering a robust immune response that prevents disease and confers resistance. In the absence of this recognition—due to insufficient or absent R gene products—the pathogen evades detection, becomes virulent, and establishes a compatible, disease-causing interaction. Effector-triggered immunity frequently results in a hypersensitive response (HR), characterized by localized programmed cell death at the infection site [3,4].

### 2.2. Environmental Stressors

Plants face numerous abiotic environmental stresses that impact their survival and output. Abiotic stressors such as drought, salinity, severe temperatures, heavy metals, and ultraviolet (UV) radiation all provide significant difficulties to plant growth and development, demanding the activation of specialized defensive mechanisms, such as stress-responsive proteins [21,22]. These stressors affect cellular homeostasis, impede metabolic activities, and cause oxidative stress by overproducing reactive oxygen species (ROS), resulting in cellular damage. In response, plants create a variety of defense proteins to reduce stress-induced damage and maintain physiological stability. Heat shock proteins (HSPs) act as molecular chaperones to prevent protein misfolding and aggregation during temperature stress, as late embryogenesis abundant (LEA) proteins assist in stabilizing cellular structures and proteins under drought and osmotic stress conditions. Antioxidant enzymes, including superoxide dismutase (SOD), catalase (CAT), and peroxidases (PODs), are critical in scavenging excess ROS and protecting against oxidative damage. Furthermore, ion transporters and osmoprotectant-associated proteins, including aquaporins and dehydrins, contribute to water balance and ion homeostasis in saline and dry situations [23,24,25]. Recent discoveries have highlighted the role of small heat shock proteins (sHSPs) in cross-tolerance to multiple stressors, as well as the interplay between stress-signaling pathways such as abscisic acid (ABA)-mediated responses and calcium-dependent protein kinases (CDPKs), in regulating abiotic stress adaptation [26]. Thanks to genetic engineering and molecular biology developments, essential stress-responsive proteins can now be overexpressed in crops, increasing their resistance to adverse environmental circumstances [27,28,29]. With applications in sustainable agriculture and biotechnology innovations, an understanding of the dynamic interplay between environmental stresses and plant defense proteins offers essential insights for enhancing crop resilience. Biotic and abiotic stressors share common signaling elements, including abscisic acid (ABA)-mediated pathways, calcium-dependent protein kinases (CDPKs), MAPK activation, and reactive oxygen species (ROS) signaling. These components serve as convergent points between biotic and abiotic stress responses, showing the overlapping roles of defense proteins and reflecting the integrated nature of plant immune responses [30,31].

## 3. Defense Protein Categories

The pathogenesis-related (PR) proteins are a group of defense-related proteins induced in response to pathogen attack [32]. PR proteins exhibit various functions, including antimicrobial activity, protease inhibition, and cell wall reinforcement. Enzymes categorized as PR proteins include β-1,3-glucanases, chitinases, thaumatin-like proteins, proteases, peroxidases, protease inhibitors, lectins, and antimicrobial peptides and ribonucleases [32,33,34]. These proteins act together or independently to detect, counteract, and mitigate the detrimental effects of invading pathogens and pests. A comprehensive summary of these proteins and their PR categories with numerous citations is tabulated in a previous review [32].

Although many of these proteins have not been purified or examined structurally by experimental techniques, the availability of their sequences can be leveraged using AlphaFold and other advanced protein folding tools [35,36,37,38] to predict structures. Structural analyses and sequence comparisons with homologous proteins enable the identification of functional domains and motifs in plant defense proteins that are critical for their activity and regulation. For instance, analysis of the predicted structures of plant root chitinases revealed the catalytic residues involved in substrate binding and hydrolysis that were verified experimentally [39].

Chitinases hydrolyze chitin, a major component of fungal cell walls and arthropod exoskeletons. By degrading chitin, chitinases contribute to the plant’s defense against fungal pathogens and pests. Chitinases are classified into glycoside hydrolase families GH18 and GH19 based on their amino acid sequences and catalytic mechanisms. They play roles in both basal and inducible defense responses in plants [34,39,40,41].

Glucanases hydrolyze β-1,3-glucans, which are significant components of fungal cell walls. They are induced in response to pathogen attacks and contribute to plant defense by degrading fungal cell walls and inhibiting fungal growth and colonization [8].

Peroxidases reduce H_2_O_2_ or organic hydroperoxides that are produced during pathogen attack or in response to abiotic stresses such as drought [42,43,44].

Proteases are important in signaling pathways that activate events such as the hypersensitive response, where local cell death limits pathogen spread [45].

Protease inhibitors can disrupt essential physiological processes in these organisms by inhibiting proteases secreted by pathogens and herbivores, thereby contributing to plant defense. Examples of protease inhibitor families include serine protease inhibitors (e.g., Bowman–Birk inhibitors) and cysteine protease inhibitors (e.g., cystatins) [46]. An example of a potential cystatin target is the protease AvrPphB, a *P. syringae* effector that cleaves the plant kinase PBS1 protein kinase, which is specifically required for the HR response to AvrPphB mediated by the NB-LRR protein, RPS5 [47,48,49,50,51,52,53].

Lectins are carbohydrate-binding proteins that play diverse roles in plant defense, including recognizing microbial pathogens, modulating immune responses, and defending against herbivores. They are involved in plants’ basal and induced defense mechanisms and can agglutinate microbial cells, inhibit microbial growth, and induce programmed cell death in infected tissues [54]. The lectin-type domain can also be part of other receptors, such as lectin receptor-like kinases (LecRLKs) and NB-LRR, allowing them to recognize specific carbohydrate patterns on pathogens and acting as a crucial component in plant immune response mechanisms [55,56].

Antimicrobial peptides (AMPs) are small, cationic peptides that exhibit broad-spectrum antimicrobial activity against bacteria, fungi, and other pathogens [57]. Plants produce AMPs in response to pathogen attacks, and AMPs contribute to defense by disrupting microbial cell membranes, inhibiting cell wall synthesis, and modulating immune responses [4,58,59]. Examples of plant AMPs include defensins, thionins, and lipid transfer proteins.

These examples, far from a complete list, highlight the many types of proteins involved in plant defense. Effective defense against diseases, pests, and environmental challenges depends on the coordinated activity of each protein class, each performing a distinct and complementary function in plant defense. It is essential to comprehend the roles and processes of these proteins to understand how to improve plant resilience and resistance in both natural and agricultural environments.

Harnessing the potential of plant defense proteins holds promise for various agricultural, environmental, and biomedical applications. In agriculture, these proteins can be utilized to develop crop varieties with improved resistance to pathogens and pests, reducing the reliance on chemical pesticides and enhancing crop productivity. Furthermore, plant defense proteins have applications in bioremediation, biocontrol, and human health, including the development of sustainable agricultural practices, bio-based products, and therapeutics [44,60,61,62].

## 4. Defense Protein Localization

Plants primarily rely on readily available, small defensive molecules like peptides, sugars, and secondary metabolites in the apoplastic space (the area outside the plant cell plasma membrane) as their first line of defense against pathogens or environmental stressors, because it requires less energy and metabolic input compared to more complex cellular responses, allowing them to minimize stress impact before it reaches the cellular level [9,63]. In addition, many different symplastic mechanisms also provide efficient stress defense, including antioxidative enzymes, peptides, defensins, secondary metabolites, and chemical antioxidants (Table 1; Figure 1).

## 5. Examples of Known Critical Defense Proteins

### 5.1. Glycosyl Hydrolases (GHs) in Archaeplastida

Plant glycosyl hydrolases (GHs) play a critical role in the breakdown of carbohydrates and glycoconjugates, supporting various cellular processes such as pathogen defense, energy reserve mobilization, and cell wall remodeling or disassembly. Recent studies have examined the distribution of GH genes across the Archaeplastida supergroup, which includes green plants, glaucophytes, and red algae. The GH gene repertoire has expanded significantly, from a few dozen genes in early Archaeplastidas to over 400 in modern angiosperms, encompassing 40 GH families in land plants. These expansions align with major evolutionary transitions. Notably, green plants acquired at least 23 GH families—primarily via horizontal gene transfer from bacteria and fungi. As green plants evolved, GH activity became increasingly extracellular, correlating with the diversification of cell wall polysaccharides and enhanced pathogen defense strategies [92]. These findings underscore the role of horizontal gene transfer and functional diversification of GHs in plant adaptation, offering key insights into the macroevolutionary processes shaping plant genomes.

### 5.2. Jacalin-Related Lectins; Subcellular Localization

The extensive protein family known as jacalin-related lectins (JALs), derived from jacalin, is one of the many lectin protein families involved in plant defensive responses. These JALs are further divided into the galactose-specific JALs and the mannose-specific JALs based on their unique carbohydrate-binding properties [93,94]. Interestingly, mannose-specific JALs are mainly located in the cytoplasm, whereas galactose-specific JALs are found primarily in storage vacuoles. Their varying subcellular localization highlights their numerous functional roles within various intracellular compartments. Several JALs are involved in the plant defense mechanism linked to endoplasmic reticulum (ER) bodies, which are derived organelles from the ER. A variety of triggers, including tissue damage, Pseudomonas syringae infection, and the administration of exogenous methyl jasmonate (MeJA), can induce ER bodies [54].

### 5.3. Plant Defense Initiated by Chloroplast Disruption

**Figure 2:**
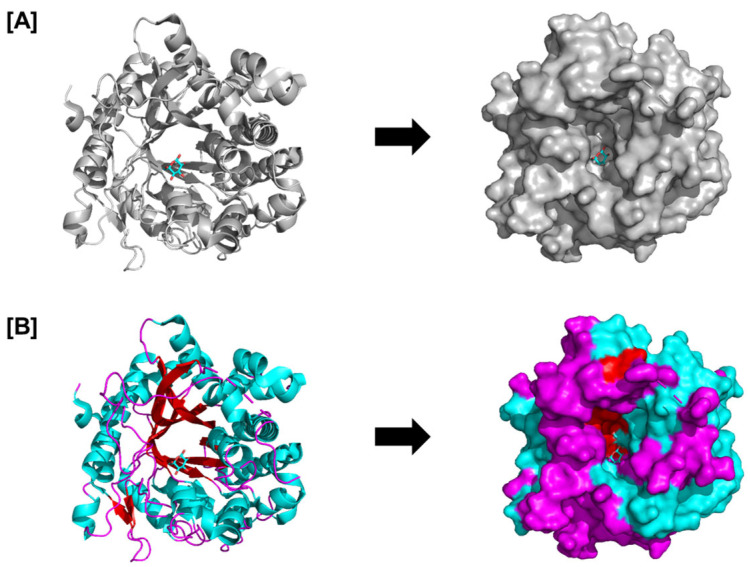
Structure of the complex between glucose and raucaffricine-O-beta-D-glucosidase; PDB ID: 4ATL. The catalytic beta-barrel is seen in (**A**) the overall glucosidase structure, and (**B**) the surface and cartoon structures display the arrangement of loops (magenta), beta sheets (red), and helices (cyan). The figure represents the structural basis of the PnGH1 enzyme involved in the two-component defense and that structure–function insights (e.g., catalytic pocket and substrate specificity) are relevant for activation post-chloroplast disruption.

Plants often produce defensive metabolites in inactive forms to minimize the risk of harm to themselves. When a pathogen attacks, these defense metabolites are spatiotemporally activated [2,9]. The so-called two-component system is essential to many plants’ chemical defense mechanisms. Ginsenosides are abundant secondary metabolites unique to species and organs in Panax plants. These ginsenosides are synthesized by *Panax notoginseng* using many resources, suggesting that the chemicals have essential beneficial roles in the plant. This crucial medicinal plant, *Panax notoginseng*, has evolved a two-component chemical defense mechanism consisting of a β-glucosidase localized in the chloroplast (Figure 2), denominated PnGH1, and its substrates, 20(S)-protopanaxadiol ginsenosides. Under physiological conditions, β-glucosidase and its substrates are spatially separated in cells; consequently, ginsenoside hydrolysis is only triggered following disruption of the chloroplast, which is brought on by the induced exoenzymes of pathogenic fungus when exposed to plant leaves. This enzyme selectively cleaves the outer glucose linked to the C-3, initiating the stimulation of PnGH1-mediated hydrolysis, which produces a range of less-polar ginsenosides with a greater spectrum of more potent antifungal activity than the precursor molecules both in vitro and in vivo. Furthermore, *P. quinquefolium* and *P. ginseng*, two congeneric taxa, are also reported to exhibit similar β-glucosidase-mediated hydrolysis with fungal infection. This discovery provides information for generating botanical insecticides to control plant diseases [95].

### 5.4. Rhizosphere-Associated Chitinases

Plant roots secrete chitinases into the rhizosphere (Table 1 and Figure 1), a complex and dynamic environment where intense nutrient exchange occurs between plants and microbes [9,96]. Root chitinases are believed to play a crucial role in plant defense against biotic and abiotic stresses because their expression is frequently upregulated in response to pathogen assaults or environmental stress. Furthermore, chitinases’ root exudation into the rhizosphere negatively affects soilborne pathogens and enhances plants’ general resilience and health. Moreover, the activity of chitinases can help root-associated microorganisms like rhizobacteria and mycorrhizal fungi, which use chitin as a source of nutrients, develop symbiotic relationships with plants for increased disease resistance [39,96]. Chito-oligosaccharides or chitin oligomers released by the action of plant chitinases are well-studied examples of PAMPs involved in defense mechanisms [97].

**Figure 3:**
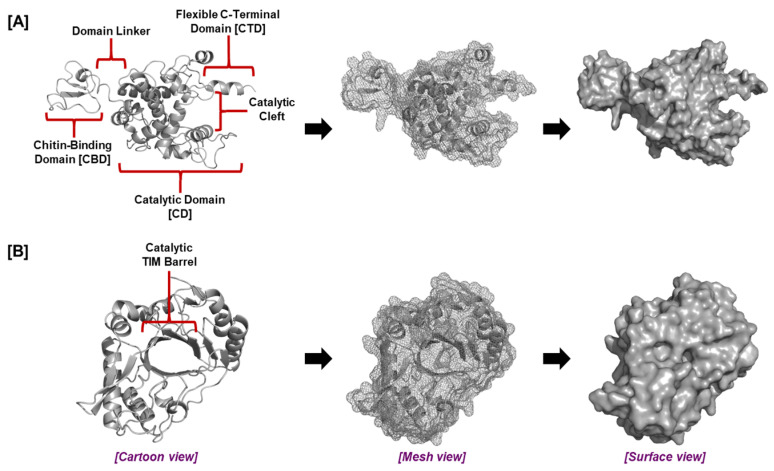
The general structure of plant root chitinases. The tertiary structure and domains of (**A**) GH19 and (**B**) GH18 chitinases.

The roles of *Zea mays* and *Oryza sativa* root chitinases, together with the chitinase of the plant root symbiotic bacterium, *Chitinophaga oryzae* in plant–microbe interactions in the rhizosphere have been determined through structural and functional characterization [39]. *Zea mays* basic endochitinaseA (*Zm*Chi19A) and *Oryza sativa* chitinase (*Os*Chi19A) belong to the glycoside hydrolase GH19 family (Figure 3A). In contrast, the *Chitinophaga oryzae* 1303 chitinase (*Csp*Ch18A) belongs to the GH18 family (Figure 3B) [39]. All GH18 chitinases are characterized by a catalytic region that consists of a triosephosphate isomerase (TIM) barrel (β/α) domain, while the catalytic domain of family GH19 is an α-helix rich lysozyme-like domain characterized by a deep cleft [98].

Modeling studies identified the presence of a flexible C-terminal domain (CTD) adjacent to the catalytic domain of the plant root GH19 chitinases which, together with the chitin-binding domain (CBD), was vital for inhibiting fungal growth (Figure 3). Both the CBD and CTD are required for optimal chitinase activity [39,99,100,101]. The effectiveness of chitin breakdown in the soil may be increased by the CTD, which may offer a second site of contact with chitin in addition to the CBD or may retain chitin in the catalytic cleft for enzymatic destruction. The plant root chitinases *Zm*Chi19A and *Os*Chi19A display distinct surface charge distributions and pH optima while sharing similar structures and conserved domains; these differences may reflect adaptations to disparate environments. Rice plants are C3 plants that thrive in temperate regions, while maize plants are C4 plants [102] better adapted to hot climates [103,104]. *Zm*Chi19A and *Os*Chi19A differ in surface charge distributions and overall surface charge. These enzymes also differ in the conditions (pH, temperature) required for optimal activity. The observed differences are likely to be relevant to the different environments into which these chitinases are released, with *Oryza sativa* usually grown in water and *Zea mays* grown in more arid settings on land [105]. Thus, these molecular variations might represent adaptations to different ecological niches, lifestyles, or host–pathogen interactions [106,107,108].

## 6. Protein Functions in Defense

Recent reports have evidenced significant progress in our knowledge of the complex roles that plant defense proteins play in modulating plant immunity and stress responses. These studies have expanded our understanding of the various roles that plant defense proteins play, highlighting their essential involvement in immunity, stress reactions, and crop adaptability.

### 6.1. SWEET Proteins

SWEETs (Sugars Will Eventually be Exported Transporters) are a newly identified family of sugar uniporters [91,109,110]. They mediate between concentration gradients and the solute potential-driven influx or efflux of sugars across the membrane. Genome-wide identification of SWEETs from various species and additional in-depth functional analyses have indicated the essential roles of the encoded proteins in modulating multiple physiological processes and enhancing plant stress resistance. SWEETs are key actors in plant–pathogen interaction because they promote the efflux of sugars and hence are very vulnerable to pathogen hijacking [111]. Furthermore, neither the virus nor the host can completely absorb sucrose, and little is known about how SWEETs affect the host or pathogen’s ability to metabolize sugar. SWEETs are involved in carbohydrate translocation and regulating abiotic stress tolerance in plants. In addition to their roles as S genes in plant–pathogen interactions, they have also been shown to facilitate an understanding of the intricate dynamics of carbohydrate metabolism during plant–pathogen interactions [63,91]. Engineering these genes could be valuable for increasing crop productivity by improving source and sink strengths and creating disease-resistant crop varieties.

### 6.2. Pathogen-Related Proteins (PR) Are Pivotal in Plant Defense

In plant defense, pathogen-related (PR) proteins are essential for battling a variety of biotic and abiotic stressors. Molecular docking, phylogenetic analysis, and molecular characterization from real-time and transcriptome investigations identified three PR-1 genes from *Musa* spp. that showed considerable upregulation in resistant cultivars when exposed to the fungal pathogens such as *Fusarium oxysporum* and *Pseudocercospora eumusae* and nematodes such as *Pratylenchus coffeae*. The PR-1 genes have distinct properties: Groups 1 and 2 are basic proteins without signal peptides, whereas Group 3 is acidic with signal peptides. The results highlight how vital PR-1 genes are to plant defense systems against biotic stressors [112]. Another study on a diverse family of PR proteins called thaumatin-like proteins analyzed two genes, *VfTLP4-3* and *VfTLP5*, and showed them to be critical for the faba bean’s response to drought stress at the seedling stage. These genes may regulate certain metabolic pathways to mediate the drought response in faba beans and provide a basis for future genetic improvement efforts to enhance drought resistance in this commercially significant crop [34].

### 6.3. BAHD Acyltransferase in Herbivorous Pest Control

Plant proteins from the BAHD acyltransferase family can acylate a range of primary and specific secondary compounds. The BAHD acyltransferase family was named according to the first letter of each of the first four biochemically characterized enzymes in this family: benzylalcohol O-acetyltransferase (BEAT), anthocyanin O-hydroxycinnamoyltransferase (AHCT), anthranilate N-hydroxycinnamoyl/benzoyltransferase (HCBT), and deacetylvindoline 4-O-acetyltransferase (DAT) [89]. Plant growth and development, fragrance generation, and biotic and abiotic stress responses are significantly influenced by the BAHD acyltransferases, which are a prominent metabolic protein domain family in terrestrial plant genomes. Comparative genomic analyses suggest that the BAHD acyltransferase gene family has undergone significant expansion during the evolutionary transition from Assam-type (*Camellia assamica*) to sinensis-type (*Camellia sinensis*) wild tea plants [88]. Phylogenetic evolution studies show that all seven clades of this family in angiosperms are present in tea plants, indicating their functional diversity. The tea plant BAHD family contains many light-, hormone-, and stress-responsive elements, expressed mainly in buds or young leaves. Some members of the BAHD family were verified for different types of herbivorous pest-feeding responses using qPCR screening and were shown to respond significantly to both pricking and chewing pests. Through their acylation to aromatic alcohols, they may participate in the defensive response of tea plants, and several TFs may also control this biological activity. This establishes a foundation for investigating the functions of BAHDs in tea plants in defensive mechanisms. Understanding the BAHD family will improve our understanding of plant ecology, given the family enzymes’ promiscuity and the speed at which neofunctionalization has evolved. Furthermore, these broad acylated alterations could be helpful in human healthcare and economic growth [88,89].

**Figure 4:**
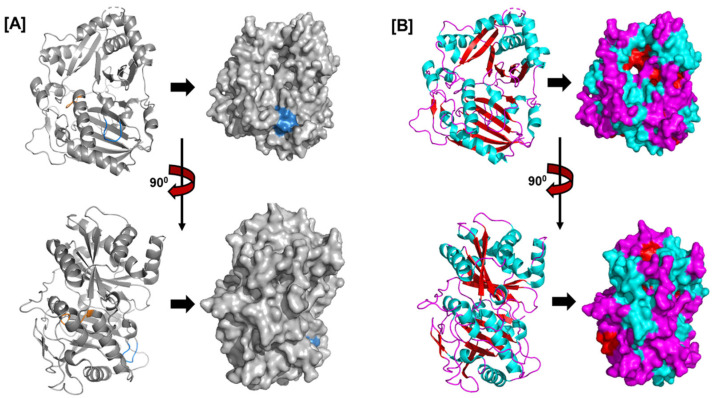
The structure of *Rauvolfia serpentina*’s vitamin synthase (PDB ID: 2BGH), a member of the BAHD acyltransferase family. The structure shows the (**A**) HXXXD (orange) and DFGWG motifs (blue) and the (**B**) arrangement of helices (cyan), beta sheets (red), and loops (magenta).

Plant BAHDs have diverse fundamental structures; at the amino acid level, several sequences belonging to distinct clades only exhibit 10–30% commonality. Conversely, up to 90% of functionally comparable members from different species show resemblance when compared pairwise, such as having proteins that produce methyl esters of benzoic acid. Even though BAHD proteins differ widely in their structures, they all have two conserved motifs, DFGWG and HXXXD (Figure 4A), and their molecular masses cluster in a range from 48 to 55 kDa, with an average of 445 amino acids [88,89]. The HXXXD motif is critical for catalysis (acting as a general base), while the DFGWG motif is important for CoA binding and proper substrate positioning during acylation reactions [88,89].

The HXXXD motif, which is conserved in BAHD acyltransferases and is crucial for catalysis, is situated close to the center of each enzyme. The DFGWG motif close to the protein’s C-terminal may be critical to catalysis and CoA binding. The spatial topologies of BAHD members are comparable despite their variances in order. *Rauvolfia serpentina* vinorine synthase (RsVS) (PDB ID: 2BGH), a globular protein with two nearly equal-sized domains joined by a crossover loop (amino acids 201–213) made up of 13 α-helixes (α1–α13) and 14 β-strands (β1–β14), was the first crystal structure of a BAHD member to be characterized (Figure 4B) [89].

Future research on BAHD acyltransferases should prioritize their biochemical characterization. Developing effective in vitro and in vivo analytical systems for functional analysis and investigating substrate specificity will be crucial in advancing our understanding of their donor and substrate preferences. These insights are expected to enhance the application of BAHD acyltransferases in metabolic engineering and synthetic biology, enabling more precise and efficient biotechnological innovations.

### 6.4. JALs in Protein Recognition and Defense

Plant lectins are a unique class of proteins extensively investigated to clarify their biological roles in signal transduction, innate immunity, and plant defense mechanisms. They specialize in recognizing and binding carbohydrates. A distinct class of plant lectins called jacalin-related lectins (JALs), descended from the jacalin protein family, is involved in crucial aspects of plant defense responses [93,94]. A recent study provides valuable insights into the interacting protein network and biological function of JAL30, for which expression is correlated with wounding and bacterial stimuli, suggesting potential involvement in the jasmonate (JA) response and ETI. Moreover, MeJA treatment increases the expression of JAL30, underscoring its anticipated role in plant defense systems. Consistent with its categorization as a lectin, JAL30 binds the O-GlcNAcylated protein ESM1, which suggests that JAL30 might also bind glycosylated pathogen-associated molecular patterns (PAMPs), such as fungal β-glucans and bacterial lipopolysaccharides [54,94].

### 6.5. DNA Binding Proteins

The vast family of proteins known as DNA-binding with one-finger (Dof) proteins regulate the expression of genes sensitive to stress or interact with other regulatory proteins to play essential roles in stress tolerance. Dof is a traditional transcription factor family unique to plants and distinguished by having a single zinc finger. Recent advanced taxonomic research on many species has revealed that numerous Dof proteins are essential to the plant life cycle [113]. Developing seed and plant resistance against various biotic and abiotic challenges depends on Dof transcription factors. Based on the presence of conserved motifs, domains, and phylogenetic analysis, 26 members of the Dof gene family were renamed *HuDof-1* to *HuDof-26* and grouped into seven subfamilies. Numerous cis-acting elements linked to phytohormones, including gibberellin, temperature, light, jasmonic acid, and abscisic acid, are found in the promoter regions of HuDof genes. Three potential candidate genes (*HuDof-1*, *HuDof-2*, and *HuDof-8*) that may play different roles in mitigating abiotic challenges were found by the transcriptome analysis of pitaya (*Selenicereus undatus*) plants exposed to abiotic pressures. This work offers a theoretical framework for functional analysis using conventional and cutting-edge biotechnological techniques to enhance agricultural traits [86].

### 6.6. Plant Defense Proteins in Viral Resistance

Plant viral infections can spread through various channels, leading to serious crop diseases and significant financial losses. Successful viral infection of a plant produces a variety of visible signs, such as disease spots, necrosis, deformities, abnormally pigmented flowers and leaves, morphological abnormalities, ulcerations, short stature, and stunted development. Presently, plant viral disease prevention involves the use of chemical pesticides, biological control techniques, and agricultural control approaches. Chemical insecticides are used to suppress insect vectors (e.g., aphids, whiteflies) that transmit plant viruses between hosts. However, they are ineffective against viruses transmitted through seed or mechanical contact and do not act directly on viruses [114,115]. Furthermore, excessive pesticide use can stress plants and disrupt ecological balance, but viruses themselves do not develop resistance to these chemicals [116,117,118]. Managing viral infections by genetic resistance in crops is a highly effective and potentially long-lasting approach [90,113,119].

Zinc finger proteins are a broad class of transcription factors that play a role in plant biotic and abiotic stress response mechanisms [120]. A study investigating the antiviral function and regulatory mechanisms of the zinc finger protein-coding gene NbZFP1 in *Nicotiana benthamiana* found that its expression was upregulated as part of the salicylic acid-mediated defense response, suggesting that NbZFP1 could enhance viral resistance in tobacco and may be a valuable target for genetic engineering in crop improvement [90]. Although NbZFP1 gene upregulation suggests a role in defense, functional validation—such as gene knockouts or overexpression—is necessary to confirm their causal role in resistance.

### 6.7. Class III Plant Peroxidases

Peroxidases can be categorized as heme or non-heme enzymes and are found in both higher and lower plants. Heme peroxidases make up around 84% of all known peroxidase enzymes, according to the RedOxiBase database. Plant peroxidases classified as class III use a common (Poulos–Kraut) catalytic mechanism to breakdown hydrogen peroxide and have comparable three-dimensional structures. Reactive oxygen species (ROS), hormone production and breakdown, fruit development, defense, and cell wall construction and maintenance are all metabolized by these classes of peroxidases [42,71]. The Poulos–Kraut mechanism is a well-established two-electron oxidation–reduction pathway, characterized by distinct and sequential oxidative and reductive stages. Harnessing plant antioxidant systems, particularly peroxidases [121,122], may facilitate the development of transgenic crops with enhanced resilience to a broad spectrum of environmental stressors [44,71]. Class III peroxidases can be overexpressed in transgenic crops to enhance ROS-scavenging capacity, improving tolerance to both biotic and abiotic stress. For example, overexpression of stress-responsive genes increases resistance to several biotic and abiotic stressors [123,124,125,126,127], suggesting a resilience mechanism via controlled ROS homeostasis and cell wall reinforcement.

### 6.8. Root Chitinases

Chitinases are part of a plant’s innate immune system (Table 1; Figure 1). A primary role of chitinases’ primary roles is hydrolyzing chitin polymers into smaller oligosaccharides, such as chito-oligosaccharides (COs). As signaling molecules, COs initiate various plant defensive reactions, such as the production of antibacterial chemicals, the activation of genes linked to defense, and the deposition of lignin and callose to reinforce cell walls. Additionally, chitinases can directly impede fungal growth by interfering with the development of chitin-rich structures necessary for fungal survival and virulence. Plant root chitinases from maize (*Zea mays*), barley, rubber (*Hevea brasiliensis*), Serratia marcescens, *Aspergillus niger*, rice (*Oryza sativa*), and the toxic weed *Ipomoea carnea* showed apparent K_M_ values in the range of 3–500 µM [39,128,129,130,131,132,133]

Plant root chitinases are seen as key players in plant pathogen defense [134], and along with bacterial chitinases, they may play a role in nutrient recycling in the rhizosphere. Structural aspects of chitinases that are important for their activity have been explored for their actions on soluble and colloidal chitins. For example, removal of the CBD from the maize chitinase did not affect its cleavage rate of 4-MU-GlcNAc3, a soluble chitin substrate, but the loss of the flexible C-terminal domain reduced it (Figure 3). In contrast, the CBD and the flexible C-terminal domain of the *Zea mays* chitinase were both essential for degrading colloidal chitin and for preventing growth of *Aspergillus niger* [39]. The findings identified structural components of the *Zea mays* chitinase that are necessary for its action on chitin exoskeletons and support the role of plant chitinases in defense against pathogens having chitin exoskeletons, such as fungus.

### 6.9. G Proteins

The Gα, Gβ, and Gγ subunits of heterotrimeric G proteins are involved in innate immunity and numerous defense-related responses, among other cellular functions [3,135]. Plant G proteins are capable of nucleotide-independent activity without dissociation. Programmed cell death is an essential component of plant immunity because it prevents infections from spreading and enables the plant to defend parts that are not afflicted. To control cell death and balance tissue loss and overall plant health, plants have evolved a sophisticated network of signaling channels and molecular interactions with pathogens [135]. Using overexpression strategies, a positive regulatory mechanism for the advancement of PCD in tomatoes was revealed. PCD was significantly increased but not initiated when the XLG2 subunit of heterotrimeric G proteins alone or the Gβ and Gγ subunits were simultaneously overexpressed. Thus, although the Gβγ dimer enhances and maintaining the course of cell death, it is not the causative agent [61].

Gβ knockout resulted in strong autoimmune reactions, making it impossible to evaluate any benefits using traditional loss-of-function mutations. These findings imply that G proteins may participate in two independent processes that control various facets of plant cell death. While one of these pathways appears to be closely linked to the start phase of PCD, the other enhances defensive responses to pathogens and positively modulates PCD development [61].

Studying G protein signaling across various species is imperative to fully comprehend their function. For a deeper understanding of the molecular processes behind programmed cell death, future research should be directed toward revealing new Gβγ-downstream targets in these signaling cascades.

## 7. Identification of Novel Plant Defense Proteins Through Omics Approaches

To identify novel plant defense proteins using omics approaches, researchers typically employ techniques like transcriptomics, proteomics, and metabolomics to analyze the complete set of genes, proteins, and metabolites expressed by a plant under stress conditions. This allows the identification of specific proteins that are significantly upregulated in response to pathogen attack or environmental stressors, thereby revealing potential defense proteins that might be previously unknown [136,137]. Our understanding of rhizosphere chemistry has improved because of advances in sequencing technologies that have made it easier to analyze trace chemical compounds, investigate allelochemical production, and observe plant responses [138]. The presence in the rhizosphere of CAZymes (carbohydrate-active enZymes), such as the glycosyl hydrolases and chitinases, suggests potential tripartite symbiotic relationships among plants, rhizospheric bacteriomes, and fungiomes. Such knowledge will guide the application of omics tools to enhance plant resilience to environmental stress [139].

The study of plant defense systems has been transformed by omics technologies, which have revealed the intricate molecular workings of plants’ responses to many stresses. Recent developments in omics technology have made identifying new plant defense proteins easier. Although there are limitations to each omics strategy that may affect the sensitivity or specificity of the technique, some can be overcome by integrating complementary approaches. However, the size and complexity of omics data demand advanced analytical methods [58].

To better understand how plants respond to stressors, cutting-edge molecular technologies and artificial intelligence (AI) are combined in plant defense research through AI-assisted omics approaches. Deciphering intricate gene, protein, and metabolite networks has been a problem for traditional research; however, AI-supported omics techniques can provide creative answers. These methods improve our understanding of plant defense mechanisms by identifying critical elements in defense pathways, finding biomarkers, and exposing hidden patterns. Comprehensive knowledge may be gained by integrating many omics data sources. As AI techniques advance, they may speed the creation of crops that are resistant to stress, improve farming methods, and guarantee sustainable food [140,141,142].

## 8. Applications of Plant Defense Proteins

### 8.1. Agricultural Applications

Many agricultural applications using plant defense proteins have been developed to increase crop protection, disease and insect control, and biotic stress tolerance. They can be applied to developing genetically engineered crops that are more resistant to pests and diseases. Fungal infections cause a variety of diseases in plants and food crops after harvest that result in significant crop losses. The most popular method of treating these diseases is using chemical-based fungicides. However, these treatments affect the environment, can endanger human and animal life, and can create resistant fungus strains. Therefore, there is a pressing demand for a wide range of ecologically safe and efficient agricultural fungicides that are also favorable to the environment. The plant innate immune system has developed a variety of defense mechanisms against fungal infections, including releasing soluble proteins with broad antifungal properties that are achieved by various mechanisms [39,60].

### 8.2. Applications in Biotechnology

Plant defense proteins function as immunological modulators, natural preservatives, and antimicrobials and may be used in biotechnology to create improved foods, biopharmaceuticals, and biobased goods. Transgenic plants with increased disease and pest resistance can be created using identified defense proteins [4,58,86,87].

Because plant peroxidases can oxidize a wide variety of phenolic and non-phenolic compounds in the presence of hydrogen peroxide, they have excellent biotechnological potential in the fields of medicine, immunology, bioremediation, textile industry, polymer synthesis, and biosensor development. Additionally, plant peroxidases may be used to create methods for quickly and effectively detecting hydrogen peroxide in biological and industrial samples. Because it can create stable chromogenic products, horseradish peroxidase (HRP) is highly utilized for analytical methods and is often the enzyme of choice for enzyme-conjugated, antibody-based detection and diagnostic protocols [44].

### 8.3. Environmental Applications

Potential uses for plant defense proteins include environmental protection and bioremediation. They can lessen the need for chemical pesticides, support sustainable agriculture, and improve the cleanup of damaged soils and waterways. Crop production may be harmed by the increasing frequency of late spring and early autumn flash freezes [143]. Enhancing plants’ tolerance to cold temperatures, combined with strategies such as improving photosynthetic efficiency to increase environmental sustainability, can support crop adaptation to a changing climate.

Improved cold tolerance allows for a wider variety of growth environments and seasons while lowering the percentage of crops lost to frost damage. Although it has been challenging to find reliable cold tolerance genes, the discovery of LTT1 provided a means of developing plants with higher cold tolerance by mechanisms that do not interfere with the development of tapetum and pollen. Increased cold tolerance during the booting stage (prior to floral emergence) was achieved by a single point mutation in the rice LTT1. Cold-induced ROS production during booting was reduced in ltt1 mutant plants [144]. Similarly, the ectopic expression in tomato of the cold response protein 1 (BOCRP1) from *Brassica oleracea* increased resistance to low temperature stress. As for ltt1, this was accomplished by lowering the cold-induced ROS and increasing the clearance of H_2_O_2_ in *BoCRP1* transgenic lines, which also showed enhanced expression and activity of ROS-detoxification enzymes—superoxide dismutase (SOD), ascorbate peroxidase (ASX) and catalase (CAT) [145]. These findings guide future research to enhance crops’ ability to withstand low temperatures by suppressing ROS levels without impairing their typical growth and development [62].

## 9. Future Perspectives

Advancing our understanding of plant–microbe interactions and the role of plant defense proteins in rhizosphere ecology can significantly benefit agriculture. These insights can lead to reduced reliance on chemical pesticides and fertilizers, improved nutrient cycling, and the development of transgenic crops with enhanced defense protein activity as a sustainable alternative to chemical inputs. For instance, increased expression of chitinases in transgenic crops can bolster resistance to fungal infections. Chitinases can also serve as key components in biofungicides. Additionally, root chitinases show promise as diagnostic indicators for monitoring soil health and disease dynamics in agricultural environments [146].

In this review, we explored the roles of several plant defense proteins, including the BAHD acyltransferase family, chitinases, peroxidases, and G proteins, in plant defense mechanisms. As research continues to uncover new defense proteins, a more comprehensive understanding of the complexity of plant responses to biotic and abiotic stress is emerging. Further investigation into the mechanisms of these proteins will provide valuable insights for developing improved crops with enhanced resistance to evolving pathogens and environmental challenges, particularly in the face of climate change

A persistent challenge in agricultural biotechnology is the evolution of insect resistance to defense proteins in genetically modified crops. The history of *Bacillus thuringiensis* (Bt), an insecticidal protein widely used in transgenic crops, offers valuable insights. Introduced into corn and cotton in the late 1990s and later incorporated into soybeans, potatoes, and eggplants, Bt has provided effective pest control with minimal environmental impact and no confirmed adverse effects on humans or animals. However, the emergence of resistant insect populations has underscored the need to identify alternative insecticidal proteins with distinct mechanisms of action to sustain long-term crop protection [147,148,149].

Plant defense proteins have been used to reduce disease losses in experimental and field studies. PR-5 overexpression in banana conferred resistance to *Fusarium oxysporum* cubense (Foc), with several transgenic banana lines evaluated in containment and semi-field trials with improved Foc resistance [150]. In a different study in rice, using a constitutive expression of PR-3 rice chitinase gene, sheath blight symptoms were significantly reduced, demonstrating improved resistance to *Rhizoctonia solani* [151]. Pathogen adaptation, gene redundancy, and the limited translation of lab or greenhouse results to complex field conditions are hurdles facing the application of these defense proteins [152,153].

The success of transgenic strategies for improving disease resistance hinges not only on the choice of defense proteins but also on their precise regulation in space and time within the plant. Spatiotemporal control of gene expression allows defense responses to be deployed where and when needed, minimizing energy costs and avoiding unintended damage to host tissues. Constitutive overexpression of defense genes (e.g., PR proteins, protease inhibitors) often leads to fitness penalties, such as reduced growth, yield, or developmental delays. To address this, researchers have utilized pathogen-inducible promoters and tissue-specific promoters to drive expression only upon infection or in high-risk tissues. For example, the rice PR10a promoter has been used to control chitinase expression, enhancing resistance without affecting overall plant performance [154]. Moreover, synthetic promoters and regulatory circuits, such as those activated by phytohormone signals (e.g., salicylic acid), provide further precision [155]. The use of microRNA-regulated expression systems and translational control elements is also being explored to fine-tune protein accumulation post-transcriptionally. Despite these advances, challenges remain in predicting and engineering robust, field-relevant spatiotemporal patterns of defense expression. Environmental cues, pathogen load, and developmental stage all influence gene expression dynamics, and fine-balancing immunity and growth is still a major constraint in transgenic applications [156,157,158].

Structural biology, functional genomics, and bioinformatics are rapidly advancing fields in plant defense protein research, with powerful tools to analyze structure–function relationships. These approaches provide critical insights into the potential applications of plant defense proteins in crop improvement, paving the way for more resilient and disease-resistant agricultural systems.

An exciting future direction is the integration of AI with multi-omics data (including genomics, transcriptomics, proteomics, and metabolomics) to enable early detection of plant diseases [141]. By analyzing complex omics datasets, advanced AI algorithms will be able to identify molecular changes associated with disease onset before visible symptoms appear. Machine learning is driving significant advancements in plant genomics, enhancing our understanding of gene regulation and identifying key genes and proteins associated with resistance to biotic and abiotic stress. These developments hold great potential for improving crop quality, increasing yields, and strengthening resistance to environmental and pathogenic challenges, ultimately transforming modern agriculture.

While this review primarily references work that utilized AlphaFold 2, the recent release of AlphaFold 3 represents a significant leap forward in predicting and modeling molecular structures, not just proteins, but also DNA, RNA, ligands, ions, and small molecules in complex biological systems, presenting application potential in predicting PR protein interactions with microbial components and signaling molecules [35].

Future research on the molecular basis of plant defense will greatly benefit from advancements in diverse technical and methodological approaches across multiple disciplines. Interdisciplinary collaboration and the development of innovative strategies for integrating research methodologies will be essential for deepening our understanding of plant defense mechanisms and enhancing crop resilience against environmental and pathogenic threats.

## 10. Conclusions

Research on plant defense proteins is rapidly advancing, with important applications in biotechnology, ecology, and agriculture. While not exhaustive, this review has examined some key plant defense proteins, their catalytic mechanisms, recent discoveries, and potential applications. The diverse functions of protein classes participating in plant immune responses and their critical roles in defending against environmental stressors and pathogens highlight the complexity and sophistication of the plant immune system.

Recent discoveries in plant defense mechanisms have deepened our understanding of protein structure–function relationships, signal transduction pathways, and the regulatory networks governing plant defense protein expression and activity. These insights into the molecular foundations of plant immunity will pave the way for innovative strategies to enhance long-term crop protection and productivity, particularly through genome editing technologies such as CRISPR/Cas9.

While defense proteins hold promise for use as biopesticides, potential off-target effects on beneficial soil microbes, symbiotic organisms, and non-target insects must be carefully evaluated. Ecological studies and biosafety assessments are essential for field deployment, particularly when considering the use of AMPs, lectins, or protease inhibitors with broad-spectrum activity [159,160,161].

## Figures and Tables

**Table 1:** Characteristics of major plant defense proteins.

S/N	Class	Localization	General Characteristics	Reports
1	PR-1 proteins	Intracellular and intercellular spaces, organ, and whole plant	Homology to the superfamily of cysteine-rich proteins (15–17 kDa)	[34,39,40,41,51,52,53,59,64,65]
2	β-glucanases	Rhizosphere, Golgi, and plasma membranes	1,3-β-endoglucanase activity. Hydrolyze fungal cell wall structural 1,3-β-glucans	[8,64,66]
3	Chitinases	Apoplastic and symplastic space, organ and whole plant, rhizosphere	Glycoside hydrolase family (26–43 kDa) chitinases. Cleave cell wall chitin polymers in situ	[2,9,39,67]
4	Chitin-binding proteins	Apoplast, rhizosphere	Bind chitin (3.1–20 kDa)	[68,69,70]
5	Peroxidases	Cell wall, apoplastic and symplastic space, organ and whole plant, rhizosphere	Heme-containing monomeric glycoproteins that utilize either H_2_O_2_ or O_2_ to oxidize a wide variety of molecules	[2,9,42,44,71]
6	Thaumatin-like proteins	Apoplastic and symplastic space	Share significant amino acid homology to thaumatin. (~22 kDa) Some cause fungal cell permeability changes; others bind to 1,3-β-glucan and exhibit 1,3-β-glucanase activity	[72,73]
7	Defensins/thionins	Apoplastic and symplastic space	Low-molecular-mass, cysteine-rich proteins. Fungal inhibition probably occurs through an ion efflux mechanism	[2,9,74]
8	Cyclophilin-like proteins	Golgi, apoplastic, and symplastic space	Have peptidyl–prolyl cis–trans isomerase activity; are collectively known as immunophilins and include the FK-506-binding proteins, parvulins, and mungins	[75,76]
9	Ribosome-inactivating proteins (RIPs)	Apoplastic and symplastic space, organ, and whole plant	RNA N-glycosidases, depurinate rRNA. Inactivate fungal ribosomes in vitro and, presumably, in situ	[77,78]
10	Lipid transfer proteins (LTPs)	Rhizosphere, extracellular spaces	Molecular masses of ~8.7 kDa. Belong to the family of antimicrobial peptides (AMPs)	[2,9,58,79,80]
11	Protease inhibitors	Rhizosphere	Protein inhibitors of serine and cysteine proteases	[2,9,46]
12	Hydroxyproline-rich glycoproteins (HRGPs)	Cell wall and plasma membrane	Heavily glycosylated, includes extensins	[81,82]
13	Glutathione transferases (GSTs)	Cytosol, plasma membrane, endoplasmic reticulum, or apoplast under certain stress conditions	Play critical roles in plant defense, redox homeostasis, detoxification, and stress adaptation	[83,84,85]
14	Other proteins	Apoplastic and symplastic space, organ and whole plant, rhizosphere	Lectins, acyltransferases, SWEETs, DNA binding proteins, viridin, snakin-1, zinc finger proteins, G-proteins, flagellins	[4,33,54,58,61,63,86,87,88,89,90,91]

## Data Availability

The original contributions presented in this study are included in the article. Further inquiries can be directed to the corresponding authors.
